# Supplementary material for: Multiparametric MRI for assessment of early response to neoadjuvant sunitinib in renal cell carcinoma
Source: PLoS One. 2021 Oct 26;16(10):e0258988. doi: 10.1371/journal.pone.0258988 (PMC8547646; doi:10.1371/journal.pone.0258988)
Supplement: S1 File — (DOCX) [file pone.0258988.s001.docx]

# Supporting Methods

## Supporting MRI Technique

Axial, coronal and sagittal T_2_-weighted structural images were acquired using a respiratory-triggered fast-recovery Fast Spin-Echo pulse sequence, with these parameters: echo time (TE) 48-69 ms; repetition time (TR) 1 breath; echo train length 10-13; field of view (FoV) 35x35 cm^2^; slice thickness/gap=4/1 mm; acquisition matrix 320x224; 2 Nex with no phase-wrap to remove aliasing.

T_1_-weighted structural images were acquired using a 2D fast spoiled gradient-echo (FSPGR) sequence with these parameters: TE 4.8 ms; TR 139 ms; flip angle 70°; FoV 35x35 cm^2^; slice thickness/gap 4/1 mm; acquisition matrix 256x256; 0.75 Nex (partial Fourier); parallel imaging (ASSET) factor 2.

R2* mapping of the renal tumour was performed in sagittal orientation using a multi-echo gradient-echo sequence and ten echo times between 4.76 and 47.6 ms with 4.76 ms echo spacing. During each breath-hold, all echo times for a single slice were acquired. Other parameters were: TR 100 ms; flip angle 25°; FoV 40x40 cm^2^; slice thickness/gap 4/1 mm; acquisition matrix 128x128; receiver bandwidth ±31.25 kHz; ASSET factor 2. Two slices were acquired in each 7-second breath-hold, with multiple breath-holds used to cover the entire tumour volume.

Dynamic Contrast-Enhanced MRI (DCE-MRI) imaging data were acquired using a fast spoiled gradient-echo (FSPGR) sequence in coronal oblique orientation with the following parameters: TE 1.6 ms; TR 3.9 ms; flip angle 18°; FoV 35x35 cm^2^; slice thickness 5 mm; acquisition matrix 160x160x20–30; receiver bandwidth ±41.67 kHz; 0.5 Nex (elliptical k-space coverage); ASSET factor 2; temporal resolution 4.3–6.4 s; 94–140 dynamic phases. In each case, the total scan time was approximately 10 minutes. The number of slices (and consequently the temporal resolution) was varied to image the entire tumour volume. 0.1 mmol/kg of Gd-DOTA (Dotarem, Guerbet) was administered during the dynamic series.

The DCE-MRI acquisition was preceded by the acquisition of T_1_ mapping data using a multiple flip-angle FSPGR technique (flip angles 1°, 3°, 5°, 10°, 15°, 20°) with 1 Nex; no parallel imaging; scan time 12.5 s (breath-hold) for each flip angle, other parameters the same as for the DCE-MRI series.

## Objective sub-segmentation of viable tumour

The analyses in this manuscript were based on the manual sub-segmentation of the viable tumour after excluding cystic and necrotic components. This procedure was defined in the protocol of the NeoSun trial. However, inter-reader variability may affect manually segmented viable tumour volumes. Furthermore, manual sub-segmentation is time consuming. The following thresholding approach was implemented to investigate whether findings obtained from manually segmented sub-volumes can be replicated with automated selection of viable tumour.

Chenevert et al. have described an ADC cut-off of 1.25x10-3mm2/s for the identification of viable tumour and tumour necrosis in glioma. The same cut-off was applied to our data, identifying voxels with a D0 as viable tumour. Masks obtained from thresholding the D0 maps were transferred to the co-registered perfusion fraction maps.

In the absence of a published threshold for the differentiation of viable tissue and necrosis based on R_2_*, the 90^th^ percentile was employed as an objective parameter. Higher R2* values are associated with tissue hypoxia.

Finally, no threshold for the differentiation of viable tumour and necrosis is available for K^trans^. Therefore, a threshold on the goodness of fit of the extended Tofts model of R^2^ = 0.5 was chosen to select only voxels which are perfused well enough to obtain a reasonable estimate of the kinetic parameters. We hypothesized that this would exclude necrotic and cystic tumour components.
