# Supplementary material for: Multiparametric MRI for assessment of early response to neoadjuvant sunitinib in renal cell carcinoma
Source: PLoS One. 2021 Oct 26;16(10):e0258988. doi: 10.1371/journal.pone.0258988 (PMC8547646; doi:10.1371/journal.pone.0258988)
Supplement: S2 File — (DOCX) [file pone.0258988.s002.docx]

# Supporting Results

## Correlation of the pharmacodynamic response with imaging biomarkers

The relative reduction in K^trans^ as a marker of tumour-level pharmacodynamic response to sunitinib was correlated with the absolute value of K^trans^ (p = 0.02 ) and iAUC_90_ (p = 0.008) in the baseline examination in a post-hoc analysis. A higher K^trans^ and iAUC_90_ at baseline were predictive of a greater reduction in K^trans^ (R^2^_adj_ = 0.35 and 0.64, respectively). D0, the perfusion fraction, R2* and T1 were not correlated with the pharmacodynamic response.

## Objective sub-segmentation of viable tumour tissue

As described in the supporting methods, thresholding techniques were employed to investigate the automated selection of viable tumour. Chenevert et al. have suggested the ADC value of 1.25x10^-3^mm^2^/s as a threshold to differentiate viable tumour with lower ADC from necrosis with a higher ADC [1]. While the median D_0_ obtained from thresholding was numerically different from the manual sub-segmentation, the two values correlated strongly (r = 0.75, p < 0.001, Fig. S1a). Furthermore, responding tumours continued to show a significantly greater reduction in D_0_ following treatment and longer progression-free survival was associated with a greater reduction in D_0_ (Fig S1b and c). Hence, the manual sub-segmentation and automated thresholding were comparable in terms of their association with the clinical endpoints.

| a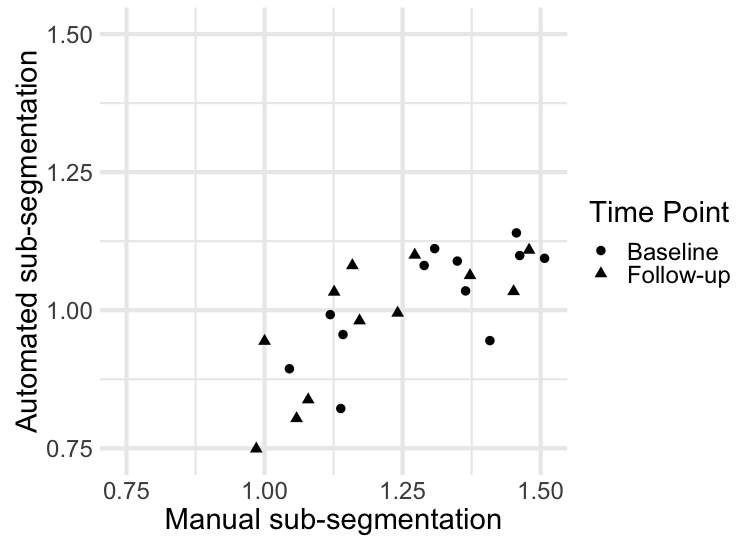 |
| --- |
| b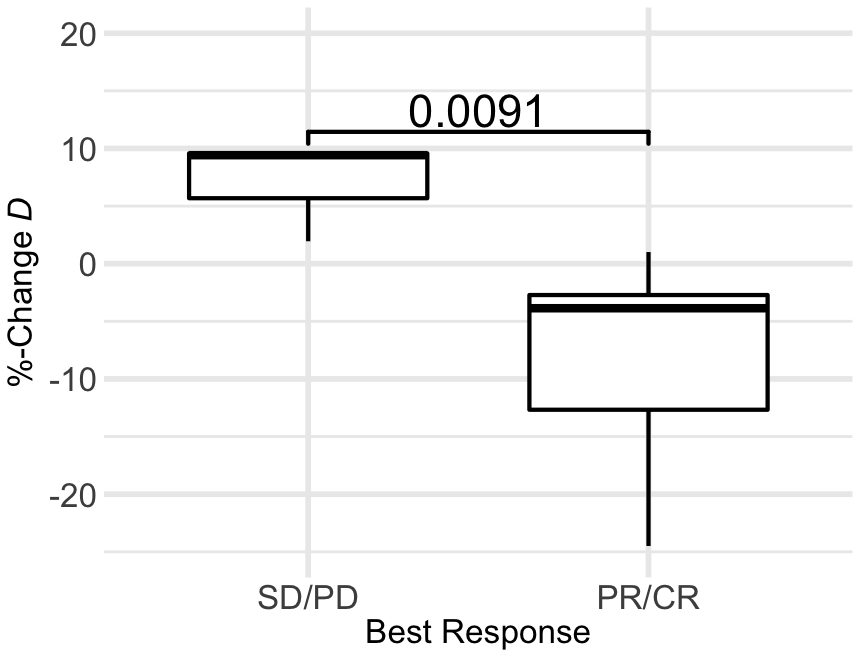 |
| c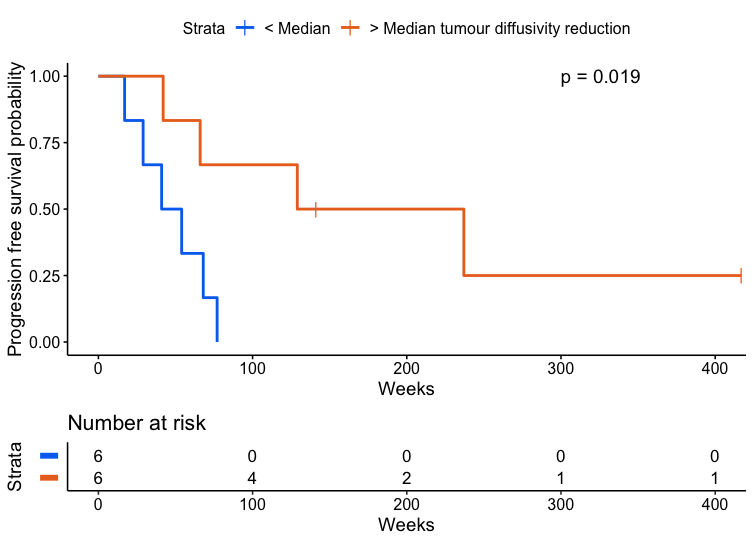  *Figure S1: a) D_0_ is strongly correlated between the manual sub-segmentation of viable tumour tissue and the automatic selection of viable voxels based on a published threshold. b) The median D_0_ derived from the automated selection of viable tumour is predictive of favourable response. c) D_0_ remains a significant predictor of progression-free survival after the automated selection of viable tumour.* |

The masks derived from thresholding the D_0_ maps as described above were transferred to the perfusion fraction maps. A strong correlation with the manual sub-segmentation was observed (r = 0.90, p < 0.001, Fig. S2). However, the perfusion fraction remained non-discriminatory between responders and non-responders and was not associated with PFS.


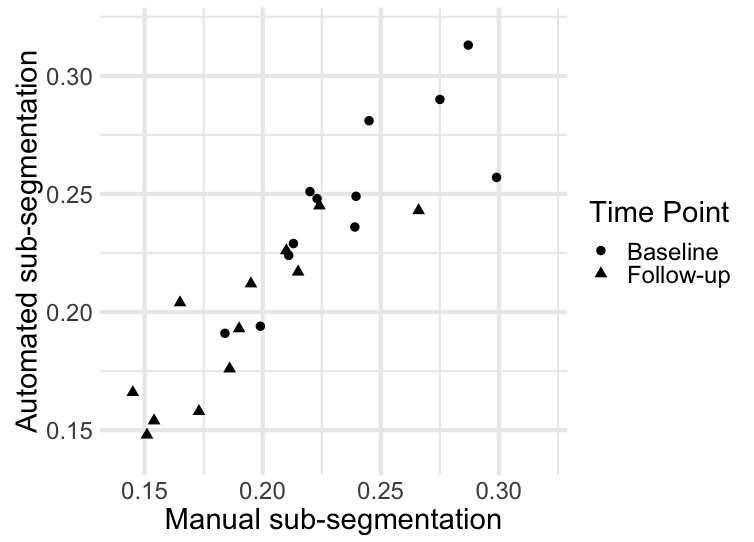


*Figure S2: The perfusion fraction is strongly correlated between the manual sub-segmentation of viable tumour tissue and the automatic selection of viable voxels based on a published threshold for co-registered D_0_ maps.*

The median tumour K^trans^ obtained through manual sub-segmentation of the viable tumour correlated strongly with the automated thresholding including only voxels where the Tofts model produced a good fit (r = 0.69, p < 0.001). However, K^trans^ remained non-discriminatory between responding and non-responding lesions and was not associated with survival.


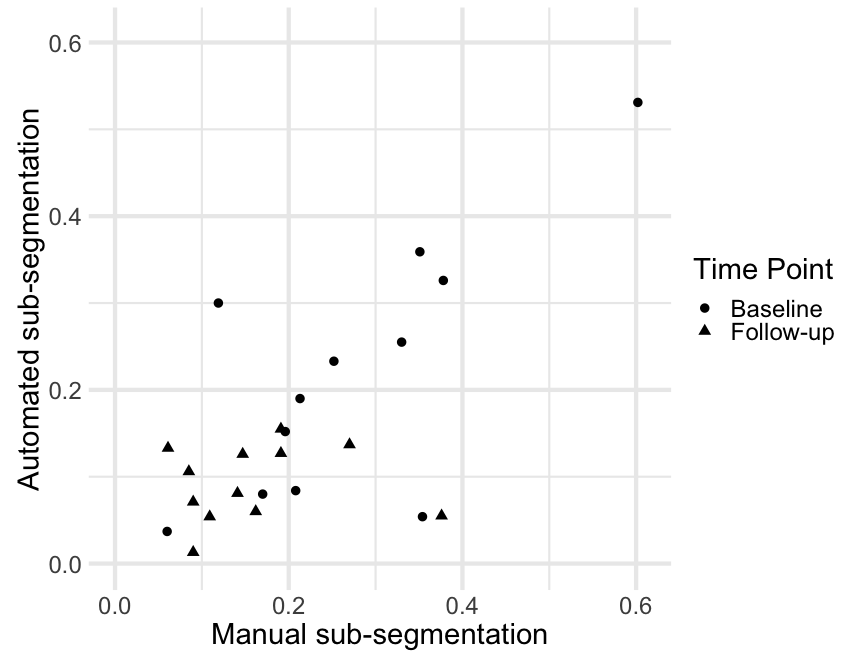


*Figure S3: K^trans^ is strongly correlated between the manual sub-segmentation of viable tumour tissue and the automatic selection of viable voxels.*

Similar to the median R2* in the viable tumour, the 90^th^ percentile of the entire lesion as an objective measure for the most hypoxic parts of a tumour increased significantly following treatment from 37 to 55 Hz (p < 0.001). However, neither the baseline value of R2* nor its change was associated with the RECIST response or survival. A comparison between the manual sub-segmentation and the thresholding at the 90^th^ percentile showed a strong correlation between the two (r = 0.94, p < 0.001).


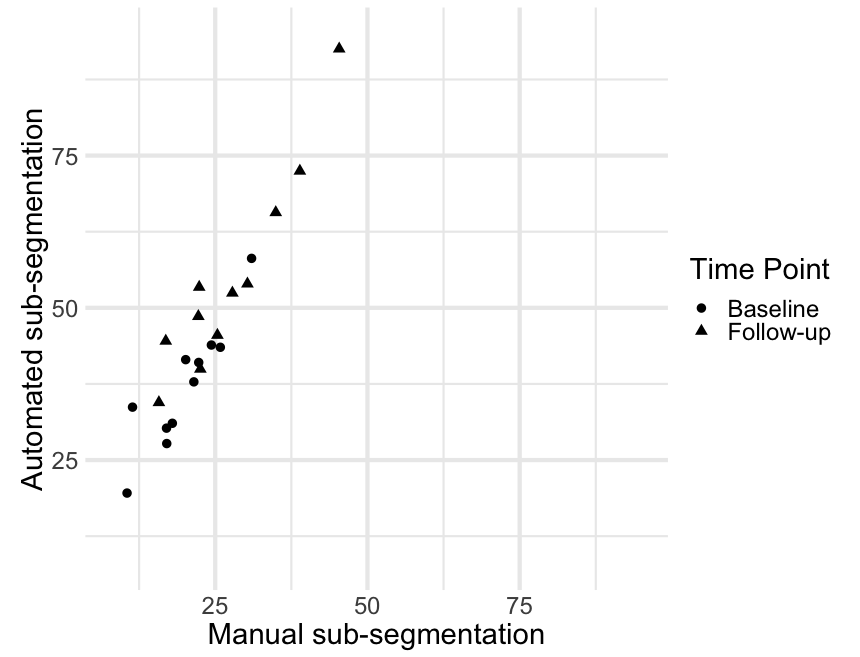


*Figure S4: R_2_* is strongly correlated between the manual sub-segmentation of viable tumour tissue and the automatic selection of viable voxels.*

1. Chenevert T, Malyarenko D, Galbán C, Gomez-Hassan D, Sundgren P, Tsien C, et al. Comparison of Voxel-Wise and Histogram Analyses of Glioma ADC Maps for Prediction of Early Therapeutic Change. Tomogr (Ann Arbor, Mich). 2019;5: 7–14. doi:10.18383/J.TOM.2018.00049
