# Supplementary material for: Multiparametric MRI for assessment of early response to neoadjuvant sunitinib in renal cell carcinoma
Source: PLoS One. 2021 Oct 26;16(10):e0258988. doi: 10.1371/journal.pone.0258988 (PMC8547646; doi:10.1371/journal.pone.0258988)
Supplement: S3 File — (DOCX) [file pone.0258988.s003.docx]

| **Patient** | **MedianADC_BL** | **MedianADC_FU** | **MedianPerfFrac_BL** | **MedianPerfFrac_FU** | **iAUC_BL** | **iAUC_F1** | **R2star_BL** | **R2star_FU** |
| --- | --- | --- | --- | --- | --- | --- | --- | --- |
| **1** | 1308 | 1126 | 239.5 | 173 | 535 | 390 | 2229 | 3890 |
| **2** | 1045 | 1172 | 239 | 151 | 581 | 424 | 1704 | 3029 |
| **3** | 1364 | 1241 | 211 | 190 | 515 | 388 | 2149 | 2536 |
| **4** | 1119 | 985 | 199 | 165 | 318 | 155 | 3097 | 4536 |
| **5** | 1289 | 1000 | 275 | 215 | 545 | 436 | 2584 | 3494 |
| **6** | 1462 | 1159 | 223 | 154 | 584 | 112 | 1600 | 2000 |
| **7** | 1456 | 1479 | 213 | 195 | 775 | 463 | 1796 | 2237 |
| **8** | 1408 | 1451 | 220 | 145 | 215 | 118 | 2015 | 2223 |
| **9** | 1349 | 1272 | 184 | 186 | 84 | 42 | 1700 | 2256 |
| **10** | 1507 | 1372 | 245 | 210 | 793 | 750 | 1052 | 1575 |
| **11** | 1142 | 1058 | 287 | 224 | 604 | 264 | 2437 | 2782 |
| **12** | 1138 | 1079 | 299 | 266 | 1084 | 772 | 1142 | 1688 |

| **Patient** | **VolSolid_BL** | **VolNorm_BL** | **VolTum_BL** | **VolSolid_FU** | **VolNorm_FU** | **VolTum_FU** | **PFSweeks** | **OSweeks** | **ObservedOS** | **ObservedPFS** |
| --- | --- | --- | --- | --- | --- | --- | --- | --- | --- | --- |
| **1** | 234.06 | 183.64 | 276.58 | 116.75 | 174.31 | 142.54 | 66 | 97 | 1 | 1 |
| **2** | 635.67 | 200.9 | 755.77 | 462.84 | 201.69 | 554.76 | 17 | 30 | 1 | 1 |
| **3** | 560.28 | 238.75 | 795.97 | 447.89 | 243.14 | 603.87 | 129 | 129 | 1 | 1 |
| **4** | 903.12 | 86.18 | 1190.3 | 749.34 | 89.02 | 953 | 141 | 374 | 1 | 0 |
| **5** | 140.82 | 238.6 | 175.42 | 84.82 | 238.11 | 116.64 | 417 | 417 | 0 | 0 |
| **6** | 194.32 | 119.73 | 204.24 | 81.46 | 129.54 | 89.63 | 29 | 71.4 | 1 | 1 |
| **7** | 615.4 | 130.67 | 753.12 | 518.59 | 138.83 | 660.73 | 77 | 146 | 1 | 1 |
| **8** | 485.15 | 150.33 | 1369.71 | 383.61 | 169.68 | 1213.4 | 41 | 126 | 1 | 1 |
| **9** | 314.71 | 326.7 | 550.83 | 269.33 | 332.21 | 463.75 | 54 | 140 | 1 | 1 |
| **10** | 296.11 | 118.55 | 629.39 | 211.14 | 118.77 | 472.43 | 237 | 326 | 0 | 1 |
| **11** | 174.14 | 257.06 | 189.99 | 132.08 | 276.09 | 146.18 | 42 | 314 | 0 | 1 |
| **12** | 103.95 | 470.74 | 701.67 | 83.05 | 475.96 | 667.72 | 68 | 311 | 0 | 1 |

**Legend:**

MedianD0_BL: D0 at baseline [x10^6^ mm^2^/s]

MedianD0_FU: D0 at follow-up [x10^6^ mm^2^/s]

MedianPerfFrac_BL: Perfusion fraction from IVIM-type diffusion weighted imaging at the baseline MRI [x10^3^]

MedianPerfFrac_FU: Perfusion fraction from IVIM-type diffusion weighted imaging at the follow-up MRI [x10^3^]

iAUC_BL: Area under the contrast enhancement curve

iAUC_F1: Area under the contrast enhancement curve

R2star_BL: R_2_* value from the baseline BOLD MRI [x10^2^ Hz]

R2star_FU: R_2_* value from the follow-up BOLD MRI [x10^2^ Hz]

VolSolid_BL: Solid tumor volume at the baseline MRI [cm^3^]

VolNorm_BL: Normal kidney volume at the baseline MRI [cm^3^]

VolTum_BL: Tumor volume at the baseline MRI [cm^3^]

VolSolid_FU: Solid tumor volume at the follow-up MRI [cm^3^]

VolNorm_FU: Normal kidney volume at the follow-up MRI [cm^3^]

VolTum_FU: Tumor volume at the follow-up MRI [cm^3^]

PFSweeks: Progression-free survival in weeks

OSweeks: Overall survival in weeks

ObservedOS: 0 = censored, 1 = OS reached

ObservedPFS: 0 = censored, 1 = PFS reached
